# Multifunctional Hollow Porous Fe_3_O_4_@N-C Nanocomposites as Anodes of Lithium-Ion Battery, Adsorbents and Surface-Enhanced Raman Scattering Substrates

**DOI:** 10.3390/molecules28135183

**Published:** 2023-07-03

**Authors:** Chunxia Qi, Mengxiao Zhao, Tian Fang, Yaping Zhu, Peisan Wang, Anjian Xie, Yuhua Shen

**Affiliations:** 1 College of Chemistry and Chemical Engineering, Anhui University, Hefei 230601, China; 2 Department of Chemical Engineering, Hefei Normal University, Hefei 230601, China; 3 School of Biomedical Engineering, Anhui Medical University, Hefei 230032, China

**Keywords:** Fe_3_O_4_@ nitrogen-doped carbon nanocomposite, hollow porous core–shell structure, multifunctional material, anode, SERS substrate, adsorbent

## Abstract

At present, it is still a challenge to prepare multifunctional composite nanomaterials with simple composition and favorable structure. Here, multifunctional Fe_3_O_4_@nitrogen-doped carbon (N-C) nanocomposites with hollow porous core-shell structure and significant electrochemical, adsorption and sensing performances were successfully synthesized through the hydrothermal method, polymer coating, then thermal annealing process in nitrogen (N_2_) and lastly etching in hydrochloric acid (HCl). The morphologies and properties of the as-obtained Fe_3_O_4_@N-C nanocomposites were markedly affected by the etching time of HCl. When the Fe_3_O_4_@N-C nanocomposites after etching for 30 min (Fe_3_O_4_@N-C-3) were applied as the anodes for lithium-ion batteries (LIBs), the invertible capacity could reach 1772 mA h g^−1^ after 100 cycles at the current density of 0.2 A g^−1^, which is much better than that of Fe_3_O_4_@N-C nanocomposites etched, respectively, for 15 min and 45 min (948 mA h g^−1^ and 1127 mA h g^−1^). Additionally, the hollow porous Fe_3_O_4_@N-C-3 nanocomposites also exhibited superior rate capacity (950 mA h g^−1^ at 0.6 A g^−1^). The excellent electrochemical properties of Fe_3_O_4_@N-C nanocomposites are attributed to their distinctive hollow porous core-shell structure and appropriate N-doped carbon coating, which could provide high-efficiency transmission channels for ions/electrons, improve the structural stability and accommodate the volume variation in the repeated Li insertion/extraction procedure. In addition, the Fe_3_O_4_@N-C nanocomposites etched by HCl for different lengths of time, especially Fe_3_O_4_@N-C-3 nanocomposites, also show good performance as adsorbents for the removal of the organic dye (methyl orange, MO) and surface-enhanced Raman scattering (SERS) substrates for the determination of a pesticide (thiram). This work provides reference for the design and preparation of multifunctional materials with peculiar pore structure and uncomplicated composition.

## 1. Introduction

Transition metal oxides (TMOs) and their related nanocomposites are widely used in the anodes of lithium-ion batteries because of their high theoretical specific capacity and excellent conductivity [1]. In recent years, their applications in other aspects have gradually attracted people’s attention, such as surface-enhanced Raman scattering [2] (SERS) and removal of pollutants [3].

Lithium-ion batteries (LIBs) are widely used in our society [4,5]. However, the commercial anode material is graphite, which cannot satisfy the ever-growing requirements of social development due to its low theoretical capacity (372 mA h g^−1^). Therefore, significant research attention has been focused on exploring the TMOs that have a higher capacity and long cycle life, such as CuO [6], Fe_2_O_3_ [7], Co_3_O_4_ [8], TiO_2_ [9] and MnO_2_ [10], etc. Among numerous alternatives, Fe_3_O_4_ has been identified as the most promising anode material for LIBs owing to its high theoretical capacity (926 mA h g^−1^), low cost, non-toxicity and abundant natural reserves [11,12,13,14]. However, its enormous volume expansion, as well as dramatic electrode pulverization, could cause serious capacity loss along with poor cycling behavior, and severely limit the practical use of Fe_3_O_4_ in LIBs; many strategies have been proposed to address these issues. Compositing with conductive matrices, such as carbon, has been proven to be a useful method to enhance electrochemical properties [15,16]. Such carbon coatings can improve the electron conductivity and relieve the volume variation during the lithiation/delithiation process. For instance, Lin et al. prepared a Fe_3_O_4_/carbon nanotube composite and confirmed a discharge capacity of 930 mA h g^−1^ after 100 cycles at 0.1 A g^−1^ [17]. In addition, some studies have demonstrated that N-doping could also increase the electronic conductivity and lithium storage properties of carbon-based materials and thus enhance their electrochemical properties [18,19]. Preparing hollow/porous and core-shell nanostructures is another efficient strategy, which could provide rapid electron channels and void spaces to accommodate the huge volume expansion of the active materials, thus improving the cycling performance of the composites. For example, He et al. synthesized porous graphene-doped carbon/Fe_3_O_4_ nanofibers, displaying a discharge capacity of 872 mA h g^−1^ at 100 mA g^−1^ after 100 cycles [20].

Protecting the human environment and reducing water pollution have always been a research focus of scientists. The direct discharge of dye pollutants such as methyl orange, methyl red and rhodamine, etc., in industry will pollute water sources and threaten human health. Therefore, the effective removal of dye pollutants in water is very important. Adsorption is commonly used in industrial wastewater purification methods. Additionally, porous Fe_3_O_4_ nanocomposite materials used as adsorbents could supply a larger surface area for adsorption, and be easily recycled using the magnetic properties of Fe_3_O_4_ [21].

Recently, SERS has attracted more attention because it is an effective detection method with fast analysis speed and favorable specificity, and therefore could be used in chemical, biological and environmental analysis. Compared with precious metal substrates with worse stability, exorbitant price and poor reproducibility, TMO substrates with low price and better chemical stability have gradually become study hotspots, such as Fe_2_O_3_ [22], Cu_2_O [23] and TiO_2_ [24]. In addition, TMO substrates with rough surfaces and porous structures can also provide more active sites for the detected molecules, thus improving the Raman response substrates [23].

**Scheme 1:**
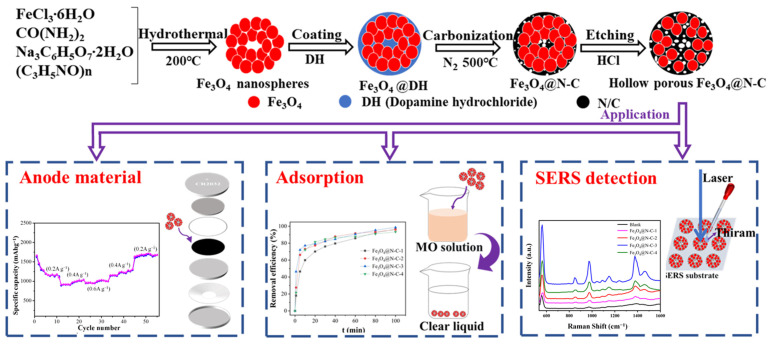
Preparation process and multi-applications of the hollow porous Fe_3_O_4_@N-C nanocomposites.

Herein, combining the above analyses, we developed a self-template mechanism for the fabrication of hollow porous Fe_3_O_4_@N-C core-shell nanocomposites (Scheme 1). Firstly, Fe_3_O_4_ nanospheres were prepared with the hydrothermal method, using FeCl_3_·6H_2_O as the raw material, Na_3_C_6_H_5_O_7_·2H_2_O as the stabilizer, CO(NH_2_)_2_ as the alkali source and (C_3_H_5_NO)_n_ as the polymer surfactant. Then, Fe_3_O_4_ nanospheres were coated with dopamine hydrochloride to form a core–shell structure, followed by calcination in nitrogen and subsequent HCl etching. Thus, the hollow porous Fe_3_O_4_@N-C nanocomposites were obtained. The influences of different etching times (15 min, 30 min and 45 min) on the morphologies and performances of the Fe_3_O_4_@N-C nanocomposites (expressed as Fe_3_O_4_@N-C-2, Fe_3_O_4_@N-C-3 and Fe_3_O_4_@N-C-4, respectively) were studied. The Fe_3_O_4_@N-C-3 nanocomposites exhibited excellent specific capacity, high-rate capacity and cycling performance as anode materials for LIBs. Meanwhile, we investigated the performances of the prepared nanocomposites in SERS detection and organic dye pollutant adsorption, obtaining the desired results. Therefore, the prepared Fe_3_O_4_@N-C nanocomposites have potential application value in energy storage, rapid SERS detection and pollutant treatment.

## 2. Results and Discussion

**Figure 1:**
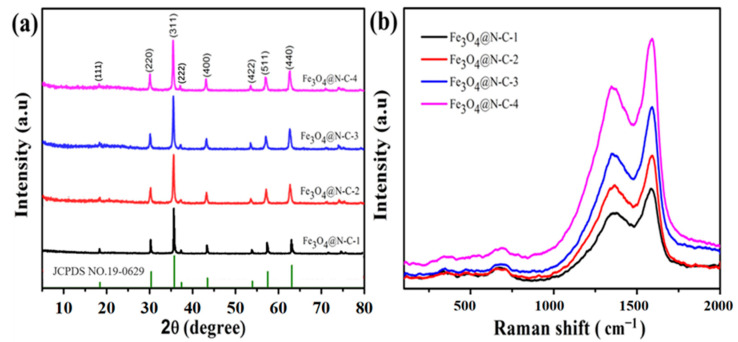
(**a**) XRD patterns and (**b**) Raman spectra of different samples.

Figure 1a exhibits the XRD patterns of the Fe_3_O_4_@N-C nanocomposites with different etching times by HCl solution. All the diffraction peaks for the four samples at 2θ of 18.22°, 30.36°, 35.77°, 37.05°, 43.35°, 53.76°, 57.16° and 62.55° could be indexed to the (111), (220), (311), (222), (400), (422), (511) and (440) crystal planes of Fe_3_O_4_ (JCPDS NO. 19-0629), respectively. No additional diffraction peaks from possible impurities, such as Fe_2_O_3_, could be detected, indicating the successful synthesis and the high purity of the Fe_3_O_4_ crystals. The sample contained less carbon; hence, the carbon peak was difficult to observe in the XRD patterns. In order to further investigate the formation of carbon on the surface of Fe_3_O_4_ and explore the change of the relative content of carbon, Raman spectra (Figure 1b) of different samples were measured. The four samples display two distinct peaks at 1352 and 1588 cm^−1^, which individually match to the D and G bands. The low-frequency D band could be attributed to structural flaws, while the G band was related to the vibration of sp2 carbon domains in both rings and chains [25]. The intensity ratios of the D band to the G band (I_D_/I_G_) of Fe_3_O_4_@N-C-1, Fe_3_O_4_@N-C-2, Fe_3_O_4_@N-C-3 and Fe_3_O_4_@N-C-4 were 0.85, 0.79, 0.78 and 0.76, respectively. With the increase of acid etching time, samples showed relatively high orderliness and graphitizing grade.

**Figure 2:**
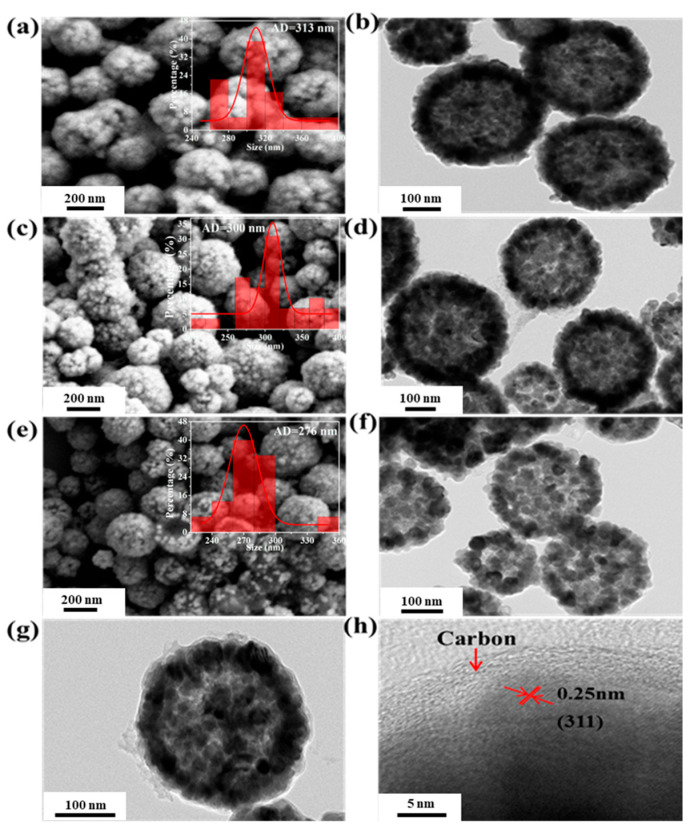
(**a**,**c**,**e**) SEM and (**b**,**d**,**f**–**h**) TEM images of (**a**,**b**) Fe_3_O_4_@N-C-2, (**c**,**d**,**g**) Fe_3_O_4_@N-C-3 and (**e**,**f**) Fe_3_O_4_@N-C-4, respectively; (**g**) high-magnification and (**h**) high-resolution TEM images of Fe_3_O_4_@N-C-3. The insets in figures (**a**,**c**,**e**) present the particle size distributions of Fe_3_O_4_@N-C-2, Fe_3_O_4_@N-C-3 and Fe_3_O_4_@N-C-4 nanocomposites.

To elucidate the morphologies and microstructures of the synthesized samples, SEM and TEM measurements were made. The SEM images (Figure 2a,c,e) show that the three samples (Fe_3_O_4_@N-C-2, Fe_3_O_4_@N-C-3 and Fe_3_O_4_@N-C-4) possess a similar spherical structure with a rough surface, and their average diameters (AD) of about 313 nm, 300 nm and 276 nm can be observed from the particle size distributions shown in the insets of Figure 2a,c,e, respectively. The sizes and morphologies of the three samples in the TEM images (Figure 2b,d,f) are consistent with those in the SEM images. The results demonstrate that with the increase of HCl etching time, the average diameter of samples gradually decreases (Figure 2b,d), and that some spherical structures of the Fe_3_O_4_@N-C-4 was even destroyed, resulting in the formation of smaller particles (Figure 2e). From the TEM images, it can be found that Fe_3_O_4_@N-C-3 nanocomposites have spherical hollow porous core-shell structures. Furthermore, the enlarged TEM image of Fe_3_O_4_@N-C-3 (Figure 2g) reveals that a N-doped carbon layer slightly increases the diameter of the sphere with a thickness of around 5 nm. Additionally, a lattice fringe with an interplanar distance of 0.25 nm is visible in the corresponding high-resolution TEM image of Fe_3_O_4_@N-C-3 (Figure 2h), which could be ascribed to the (311) plane of Fe_3_O_4_. The carbon is marked by an arrow, implying that the product consists of Fe_3_O_4_ and carbon. The hollow porous nanospheres structure can offer plentiful mesoporous channels for solid–liquid contact.

**Figure 3:**
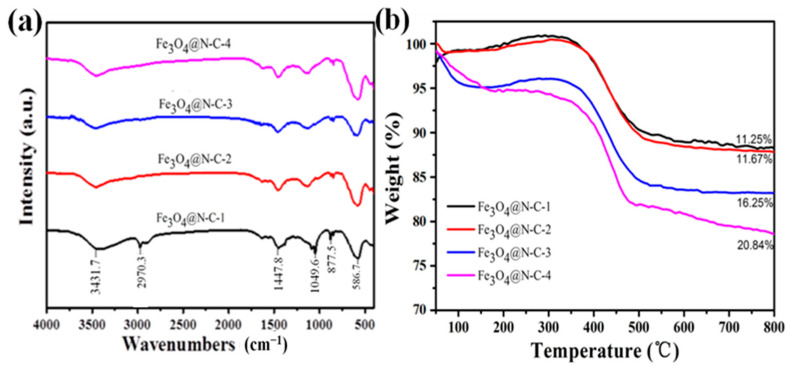
(**a**) FT-IR spectra and (**b**) TG curves of different samples.

To further prove the existence of different functional groups and the change of the peak intensity, the FT-IR spectra of the four samples were taken at a range of 400–4000 cm^−1^. As demonstrated in Figure 3a, the peaks at 3431 cm^−1^ belong to the stretching vibration of –OH while the bands appearing at 2970 and 1447 cm^−1^ belong to the C-H groups and C-N groups, respectively. The absorptions at 1049 and 877 cm^−1^ are ascribed to the C-H in-plane and out-of-plane bending vibrations of 1, 4-substituted benzene. The peak at 586 cm^−1^ is assigned to the Fe-O group in Fe_3_O_4_ [20,26]. The FT-IR measurements demonstrate the prepared Fe_3_O_4_@N-C nanocomposites contain Fe_3_O_4_ and nitrogen-doped carbon. In addition, TG analysis was implemented to estimate the carbon content in the four samples, and the results are exhibited in Figure 3b. Obviously, the four samples started to decompose at 300 °C because of the oxidation of Fe_3_O_4_ to Fe_2_O_3_. Weight loss was evident in the temperature range of 300–450 °C, which may be ascribed to carbon oxidation. Based on the TG curves, the carbon contents calculated in Fe_3_O_4_@N-C-1, Fe_3_O_4_@N-C-2, Fe_3_O_4_@N-C-3 and Fe_3_O_4_@N-C-4 were about 11.25%, 11.67%, 16.25% and 20.84%, respectively, which shows a great influence on the electrochemical performance of Fe_3_O_4_@N-C nanocomposites.

**Figure 4:**
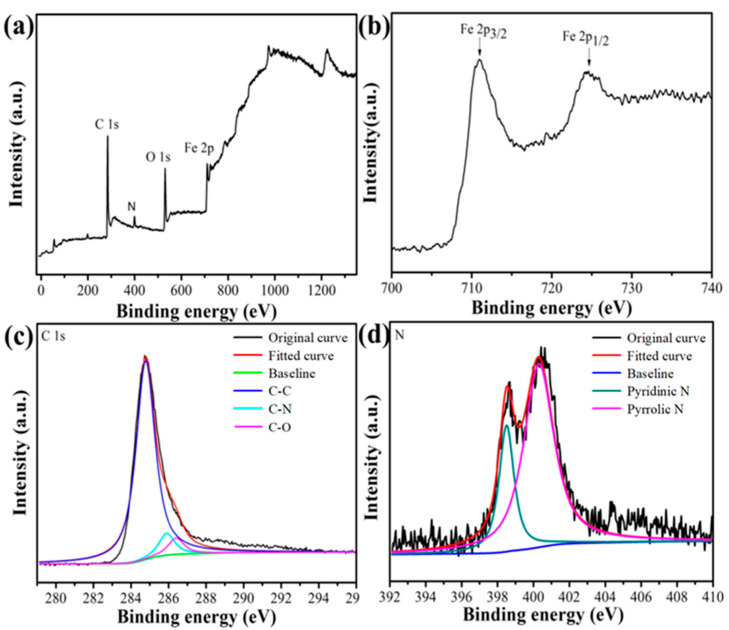
(**a**) XPS survey spectrum of the Fe_3_O_4_@N-C-3 nanocomposites, (**b**) Fe 2p, (**c**) C 1s and (**d**) N 1s core-level spectra of the Fe_3_O_4_@N-C-3 nanocomposites.

In order to determine the surface elemental composition and valence states of the Fe_3_O_4_@N-C-3 nanocomposites, XPS characterizations were further implemented. In Figure 4a, XPS analysis of Fe_3_O_4_@N-C-3 indicates that the Fe, O, C and N atoms are presented in the nanocomposites with atomic ratios of 6.05%, 17.83%, 68.75% and 7.37%, respectively. Additionally, three peaks at 285.5 eV, 400 eV, 533.5 eV and 700 eV belong to C 1s, N 1s, O 1s and Fe 2p, respectively. The high-resolution core level XPS spectra of Fe 2p [27] are exhibited in Figure 4b. There are double peaks corresponding to Fe 2p 3/2 and Fe 2p 1/2 energy level located at 710.98 eV and 724.03 eV, respectively, demonstrating the existence of Fe_3_O_4_ rather than Fe_2_O_3_ [26]. The high-resolution C 1s XPS spectrum of the Fe_3_O_4_@N-C-3 nanocomposites (Figure 4c) displays an asymmetric broad peak that corresponds to the C-C (284.68 eV), C-N (285.98 eV) and C-O (286.63 eV) functional groups on the surface, respectively, which indicates the presence of multiple chemical states of carbon and the different types of functional groups on the surface. These results indicate the formation of an N-C covalent bond and the presence of N-doped C in the nanocomposites. In the N 1s spectra (Figure 4d), the two peaks located at 400.18 eV and 398.63 eV correspond to pyrrolic nitrogen and pyridinic nitrogen groups, respectively. According to [27], the pyrrole nitrogen and pyridine structures can provide suitable channels for Li^+^ insertion.

**Figure 5:**
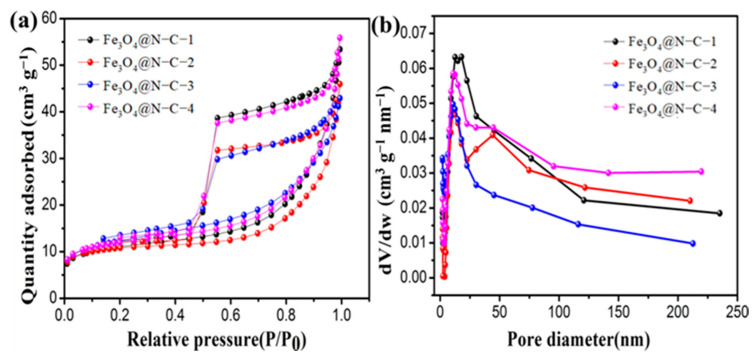
(**a**) N_2_ adsorption-desorption isotherms and (**b**) pore size distributions of different samples.

The N_2_ adsorption-desorption isotherms and the related pore size distributions calculated by the Barrett-Joyner-Halenda (BJH) method for the four samples are shown in Figure 5. The adsorption/desorption curves have a typical type IV shape (Figure 5a), corresponding to mesoporous structures with the distributions of pore size primarily around 10–20 nm (Figure 5b). The Brunauer-Emmett-Teller (BET) specific surface areas of Fe_3_O_4_@N-C-1, Fe_3_O_4_@N-C-2, Fe_3_O_4_@N-C-3 and Fe_3_O_4_@N-C-4 were calculated to be around 38, 37, 43 and 41 m^2^ g^−1^, respectively. By comparison, the Fe_3_O_4_@N-C-3 nanocomposites possessed the highest specific surface area, which can shorten ion and electron transport channels and provide sufficient contact of the nanocomposites with the electrolyte. Additionally, this special hollow porous structure can also help to accommodate the volume change during the process of charging and discharging.

**Figure 6:**
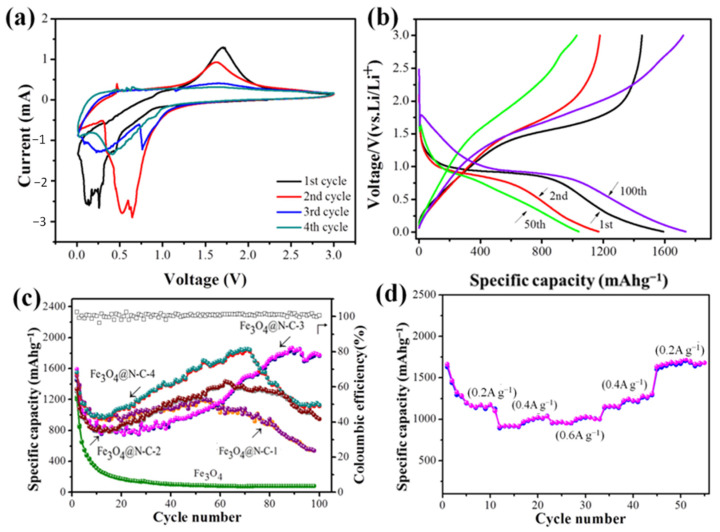
(**a**) Cyclic voltammograms at a scan rate of 0.2 mV s^−1^ and (**b**) charge-discharge profiles at 0.2 A g^−1^ of the Fe_3_O_4_@N-C-3; (**c**) cycling performance of the five samples for 100 cycles at a current density of 0.2 A g^−1^; (**d**) rate performance of Fe_3_O_4_@N-C-3 at different current densities.

The electrochemical properties of different Fe_3_O_4_@N-C samples were tested as the anode materials for LIBs. Figure 6a exhibits the first four cyclic voltammetry (CV) curves of Fe_3_O_4_@N-C-3 at a scan rate of 0.2 mV s^−1^. In the first cathodic curve, the reduction peak located around 0.54 V can be attributed to the reduction reaction of Fe^3+^ or Fe^2+^ to Fe^0^ (Fe_3_O_4_+2Li^+^+2e^−^→Li_2_Fe_3_O_4_, Li_2_Fe_3_O_4_+6Li^+^+6e^−^→4Li_2_O+3Fe), as well as the formation of a solid electrolyte interface (SEI) film [28]. In the first anodic scan, the peak at 1.75 V can be explained as the reversible oxidation of Fe^0^ to Fe^2+^ and Fe^3+^ (3Fe+4Li_2_O+8e^−^→Fe_3_O_4_+8Li). After the first cycle, the CV curves almost overlap, showing that the stable SEI film is well generated on the surfaces of the electrode material, resulting in the enhanced stability and high coulombic efficiency of the Fe_3_O_4_@N-C-3 nanocomposites.

Figure 6b presents the 1st, 2nd, 50th and 100th discharge and charge curves for the Fe_3_O_4_@N-C-3 nanocomposites in the potential range of 0.01–3.0 V at a current density of 0.2 A g^−1^. The first discharge and charge capacities of the Fe_3_O_4_@N-C-3 electrode were, respectively, 1591 and 1452 mA h g^−1^, demonstrating an initial coulombic efficiency of 91%. The loss of reversible capacity might be attributed to the disintegration of the electrolyte, development of SEI film, trapping of lithium inside the active material and some other factors [29].

The cycling performances of the hollow porous Fe_3_O_4_@N-C nanocomposites are revealed in Figure 6c. It can be seen that the discharge capacity decreases in the first several dozens of cycles and then starts to increase; an analogous circumstance has also appeared in previous reports [25,30,31]. However, the mechanism is not clear. The reasons may be as follows: (1) the improved Li-diffusion kinetics of the reactivated electrode; (2) the activation of active materials; and (3) the structure refinement and formation of a thin and stable SEI without fracture [32,33]. The Fe_3_O_4_@N-C-3 nanocomposites exhibit excellent cycling stability; a high reversible capacity of 1772 mA h g^−1^ could be acquired after 100 cycles at a current density of 0.2 A g^−1^. It should be noted that the capacity is higher than the other samples, such as Fe_3_O_4_ (79 mA h g^−1^), Fe_3_O_4_@N-C-1 (540 mA h g^−1^), Fe_3_O_4_@N-C-2 (948 mA h g^−1^) and Fe_3_O_4_@N-C-4 (1127 mA h g^−1^). Furthermore, the cycling performance of Fe_3_O_4_@N-C-3 is also higher than those of the similar products reported previously (Table 1).

Figure 6c also demonstrates the coulombic efficiency of the composites during the cycling process. The coulombic efficiency of Fe_3_O_4_@N-C-3 nanocomposite after 100 cycles could reach 99.5%, demonstrating that the nanocomposites have excellent electrochemical performance. The rate capability is a crucial feature for high-performance LIBs to measure the reversible capacity and cycling stability. In Figure 6d, the Fe_3_O_4_@N-C-3 anode has reversible capacities of 1200, 1050 and 950 mA h g^−1^ at current rates of 0.2, 0.4 and 0.6 A g^−1^, respectively. The discharge capacity of Fe_3_O_4_@N-C-3 recovered to the greater value of 1700 mA h g^−1^ when the current density returned to 0.2 A g^−1^. These findings reveal that the hollow porous nanosphere structure can preserve the integrity of electrode as the current density increases, while the outer N-doped carbon layer also prevents large volume expansion during cycling, thus exhibiting an excellent rate performance.

**Figure 7:**
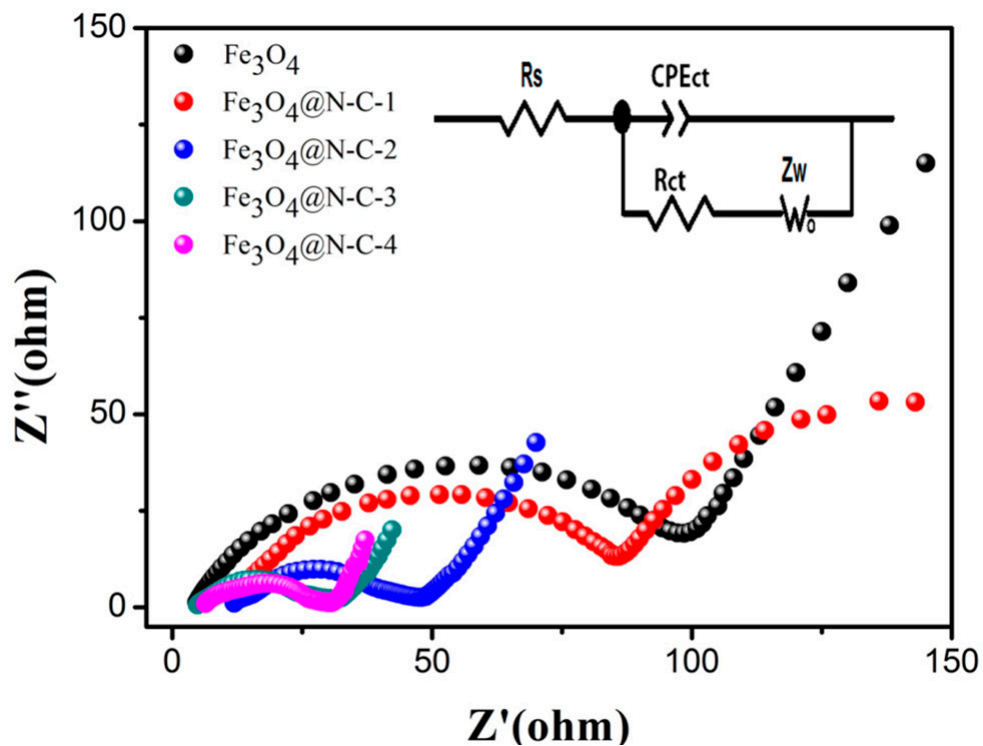
Nyquist plots of the Fe_3_O_4_@N-C electrodes in the frequency range between 100 kHz and 0.01 Hz. The inset presents the equivalent circuit.

Nyquist plots were fitted in order to better understand the character of the enhanced electrochemical performance. Figure 7 exhibits EIS spectroscopy carried out in a frequency range from 100 KHz to 0.01 Hz. The curves of five samples are all composed of a broad semicircle in the high-medium-frequency region and a long low-frequency line, which expresses the charge-transfer impedance (Rct) at the electrode/electrolyte interface and the Warburg impedance (Zw) related to the Li^+^ diffusion process in the electrode materials. The constant phase element (CPEct) represents the capacitance at the electrode-electrolyte interface. Compared to the pure Fe_3_O_4_, the electrical conductivity of the Fe_3_O_4_@N-C nanocomposites has been optimized. At the same time, the Fe_3_O_4_@N-C-3 electrode exhibits lower charge transfer resistance than other samples, which is related to the appropriate thickness of the N-C shell, high specific surface area and mesoporous structure. With these benefits, the conductivity of nanocomposites can be effectively increased, and the contact area between the electrode and the electrolyte can be expanded.

**Figure 8:**
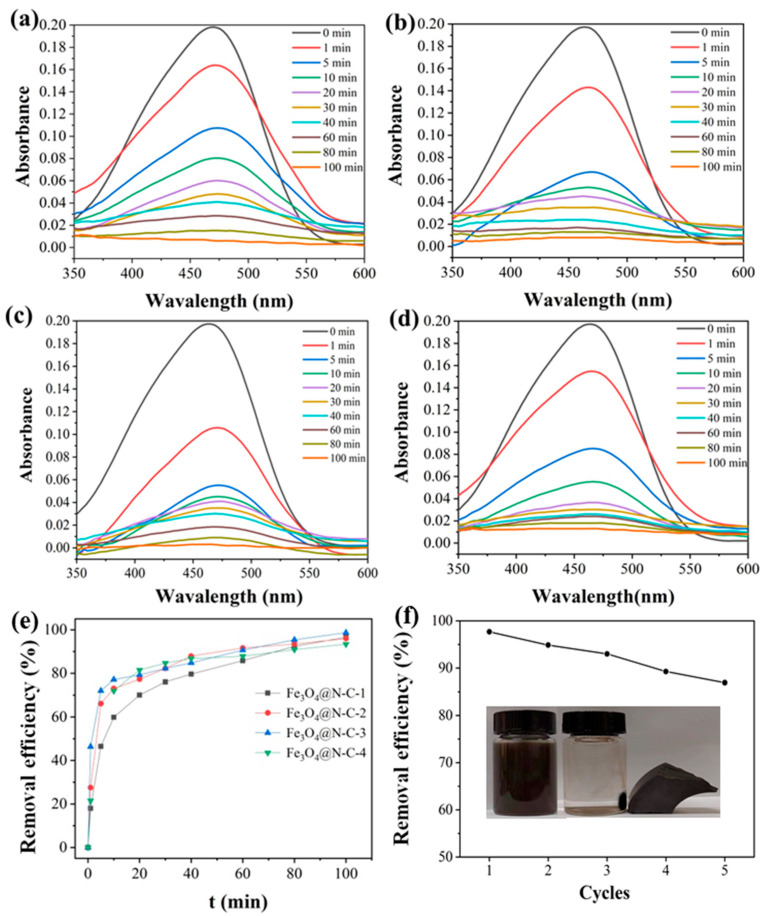
UV-Vis absorption spectra of MO over (**a**) Fe_3_O_4_@N-C-1, (**b**) Fe_3_O_4_@N-C-2, (**c**) Fe_3_O_4_@N-C-3 and (**d**) Fe_3_O_4_@N-C-4 nanocomposites. (**e**) The change of removal efficiency of MO by different Fe_3_O_4_@N-C nanocomposites with time. (**f**) The removal rate changes of MO by the Fe_3_O_4_@N-C-3 nanocomposites for five cycles. The inset shows Fe_3_O_4_@N-C-3 in water being attracted apart with an external magnet.

The adsorption capacities of Fe_3_O_4_@N-C nanocomposites to MO are shown in Figure 8a–d. It can be observed that the absorption peak intensity of MO at 465 nm rapidly declines with the extension of the adsorption duration in the presence of different Fe_3_O_4_@N-C nanocomposites. After about 100 min, the absorption peak of MO at 460 nm almost disappears. The results indicate that different Fe_3_O_4_@N-C nanocomposites all have good adsorption performances towards MO, which is due to the cooperation of the special hollow porous core-shell structure with the N-doped carbon layer [38] of the samples. From Figure 8e, we can more intuitively understand the relationship of MO adsorption behavior with time. It can be seen that the removal efficiency of MO over Fe_3_O_4_@N-C-3 at the adsorption of 100 min reaches 98.65%, while those over Fe_3_O_4_@N-C-1, Fe_3_O_4_@N-C-2 and Fe_3_O_4_@N-C-4 are 96.57%, 95.54% and 93.40%, respectively, which is due to the larger surface area increasing the contact between Fe_3_O_4_@N-C-3 and MO. The inset of Figure 8f demonstrates that the Fe_3_O_4_@N-C-3 has strong magnetism and is easy to recover and recycle. In Figure 8f, the removal efficiency of MO in the presence of Fe_3_O_4_@N-C-3 decreases to 86.92% after five cycles, indicating that the Fe_3_O_4_@N-C-3 nanocomposites as adsorbents have strong recyclability and adsorption stability, and are beneficial to the treatment of industrial dye wastewater.

**Figure 9:**
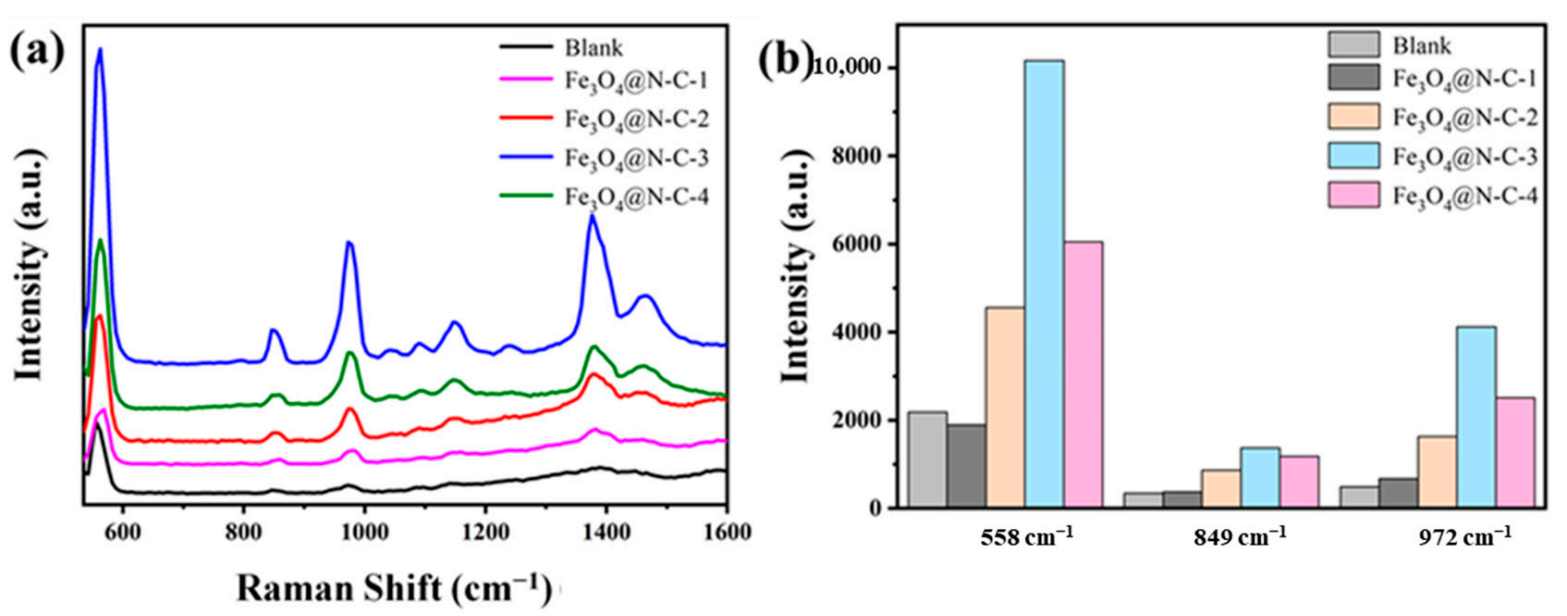
(**a**) SERS spectra of thiram before (blank) and after absorption on different Fe_3_O_4_@N-C substrates; (**b**) the related peak intensities at 558 cm^−1^, 849 cm^−1^ and 972 cm^−1^ of thiram in (**a**).

In view of the fact that Fe_3_O_4_ is a semiconductor material with good SERS property [39], therefore, we selected the pesticide thiram as the probe to detect the SERS-enhanced effects of different Fe_3_O_4_@N-C substrates. Raman spectra (Figure 9a) show that thiram on silicon substrate had a strong feature peak at 558 cm^−1^, which belongs to the characteristic band of S-S stretching vibration [40]. There are some other peaks at 849 cm^−1^, 972 cm^−1^, 1145cm^−1^, 1375 cm^−1^ and 1466 cm^−1^, which are assigned to υ (C-N-C) and υ (C-S), υ (S-C-S) and ρ (CH_3_), ρ (CH_3_) and υ (C-N), υ (C-N) and ρ (CH_3_) [41,42], respectively. Since the characteristic peak intensities of thiram after 1100 cm^−1^ could be influenced by the distinct D and G bands of carbon at 1352 cm^−1^ and 1588 cm^−1^, we chose the intensity changes of 558 cm^−1^, 849 cm^−1^ and 972 cm^−1^ peaks (Figure 9b) as the references to intuitively reflect the SERS enhancement effect of Fe_3_O_4_@N-C substrates on thiram. We found that the peak intensities of the thiram are almost universally enhanced on Fe_3_O_4_@N-C substrates, which may be on account of the hollow porosity of the Fe_3_O_4_@N-C nanocomposites enlarging the contact between the probe molecules and the substrates. It can be seen by combining Figure 9a,b, the peaks of the pesticide thiram are the strongest on the Fe_3_O_4_@N-C-3 substrate. According to BET analysis results, Fe_3_O_4_@N-C-3 has the largest specific surface area, which can offer more surface-active sites and facilitate the adsorption of probe molecules, resulting in better SERS detection performance of Fe_3_O_4_@N-C-3 for the thiram. This result indicates that the prepared Fe_3_O_4_@N-C-3 nanocomposites could be used to detect thiram with the SERS technique. Related studies will be reported separately.

## 3. Materials and Methods

### 3.1. Materials and Chemicals

Sodium citrate (Na_3_C_6_H_5_O_7_∙2H_2_O), iron (III) chloride hexahydrate (FeCl_3_∙6H_2_O, 99%), urea (CO(NH_2_)_2_) and polyacrylamide ((C_3_H_5_NO)_n_) were purchased from Sinopharm Chemical Reagent Co., Ltd. (Shanghai, China). All reagents were of analytical grade and used without further purification.

### 3.2. Synthesis of Fe_3_O_4_ Nanospheres

Firstly, FeCl_3_·6H_2_O (6.6 mmol) was dissolved into the deionized water (80 mL) under ultrasound irradiation for 20 min at room temperature to form yellow transparent solution, and then 8.0 mmol of Na_3_C_6_H_5_O_7_·2H_2_O and 12.0 mmol of urea were added to the solution under stirring for 30 min. Subsequently, 8.6 mmol of polyacrylamide was added into above solution under continuous stirring until it was dissolved totally, and a light green clear solution formed. The above solution was transferred into a 100 mL Teflon-lined stainless steel autoclave and heated at 200 °C for 12 h. The sediment was collected after being cooled down to room temperature, washed with deionized water and alcohol three times each and dried at 80 °C for 12 h to obtain Fe_3_O_4_ black powders.

### 3.3. Synthesis of Hollow Porous Fe_3_O_4_@N-C Nanosphere Composites

Overall, 0.5 g of Fe_3_O_4_ power and 0.1 g of dopamine hydrochloride were decentralized successively in 20 mL of ethanol under stirring for 30 min, and the mixture was dried under vacuum at 80 °C. The carbon-precursor-coated Fe_3_O_4_ hollow nanospheres were further heated in a tube furnace at 500 °C in nitrogen atmosphere for 4 h to acquire Fe_3_O_4_@N-C nanospheres (Fe_3_O_4_@N-C-1). In the following step, 1.0 g Fe_3_O_4_@N-C nanospheres were etched in 75 mL hydrochloric acid (HCl) solution for 15 min (Fe_3_O_4_@N-C-2), 30 min (Fe_3_O_4_@N-C-3) and 45 min (Fe_3_O_4_@N-C-4), respectively.

### 3.4. Characterization

Field-emission scanning electron microscopy (FE-SEM, S4800, Hitachi, Tokyo, Japan) and high-resolution transmission electron microscopy (HR-TEM, JEM2100, JEOL Ltd., Beijing, China) were used to investigate the morphologies of the samples. The crystal structures of the samples were determined by X-ray diffraction (XRD) (DX-2700) using Cu-Kα (30 kV, 25 mA, λ = 1.5406 Å) radiation over a 2θ range of 5–80°. A Dilor LABRAM-1B multi-channel confocal microspectrometer with an excitation wavelength of 532 nm was applied to record the Raman spectra. The surface chemical compositions of samples were investigated using X-ray photoelectron spectroscopy (XPS, ESCALAB 250, Thermo Scientific, Waltham, MA, USA). The thermogravimetry (TG) of the nanocomposites was performed in air atmosphere from room temperature to 800 °C at a heating rate of 10 °C min^−1^. Fourier transform infrared spectrometry (FT-IR) (Thermo Nicolet NEXUS 670, Thermo Scientific, Waltham, MA, USA). with a wavelength range from 400 to 4000 cm^−1^ was applied to identify the functional groups of samples. On the 3H-2000BET-A system, N_2_ adsorption-desorption isotherms were determined at liquid nitrogen temperature.

### 3.5. Electrochemical Measurements

The active material powders, acetylene black (Super P) and polyvinylidene fluoride (PVDF), were mixed in a 8:1:1 mass ratio, then added into N-methyl-2-pyrrolidinone (NMP) solvent to form a homogeneous slurry with stirring, subsequently spread on a copper foil substrate and finally dried in vacuum at 80 °C for 10 h to remove the solvent. Thus, a working electrode was obtained. The 2016 coin-type cells were assembled in an argon-filled glove box using metallic lithium as the counter electrode and polyethylene film (Celgard, 2400) as the separator. The nonaqueous electrolyte was used in the 1 M solution of LiPF_6_ dissolving in the 1:1 wt% solution of ethylene carbonate (EC) to dimethyl carbonate (DMC). Electrochemical measurements were performed at room temperature on CT-3008W-5V 10 mA battery testing equipment (Neware Technology Co. Ltd., Shenzhen, China) at various current densities ranging from 0.01 to 3.0 V. Using battery testing equipment (NEWARE BTS-3000), the galvanostatic charge-discharge profiles were determined at various current densities within the voltage window of 0.01 to 3.0 V (vs. Li/Li^+^). Cyclic voltammetry (CV) measurements were performed on an electrochemical workstation (CHI660D, Shanghai CH Instruments Co., Ltd., Shanghai, China) over the potential between 0.01 V and 3.0 V at a scanning rate of 0.2 mV s^−1^. Electrochemical impedance spectroscopy (EIS) was measured in the 100 KHz to 0.01 Hz frequency range.

### 3.6. Adsorption Measurements

As a typical pollutant, methyl orange (MO) was employed to study sample adsorption behaviors. The details are as follows: 10 mg of the sample was added to 50 mL of 10^−5^ M MO aqueous solution, and stirred at a constant speed at room temperature. At 0, 1, 5, 10, 15, 20, 30, 40, 60, 80 and 100 min, the upper clarified solution after reaction was taken out and the ultraviolet visible spectrophotometer (SHIMADZU, UV-1800, Shanghai, China) was applied to measure the absorbance of MO at 465 nm in the solution.

Through the above adsorption experiments, the sample with the best adsorption performance was selected for investigating the recycle adsorption stability. The first adsorption was carried out according to the above steps. After 100 min, the sample was separated from the solution with an external magnet, rinsed with water and ethanol three times, respectively, and then re-decentralized into 50 mL of 10^−5^ M MO aqueous solution for the adsorption experiment. The adsorption was carried out five times.

The MO removal efficiency of the sample can be computed by Formula (1):

(1) $$\mathrm{Removal}\;\mathrm{efficiency}\;(\%)=\frac{C_{0}-C_{t}}{C_{0}}\times 100\%$$

where C_0_ and C_t_ represent the MO concentration in the original and after being adsorbed by the sample for a period of time, respectively.

### 3.7. SERS Measurements

For the preparation of the SERS substrates, 10 mg of the samples was dispersed into 5 mL of anhydrous alcohol, followed by dripping a drop of the dispersions onto the silicon substrate and then natural room-temperature drying. Next, the above substrate was covered with a drop of a 10^−3^ M thiram aqueous solution, which was dried at room temperature. The blank group was obtained by dropping the 10^−3^ M thiram aqueous solution on the silicon substrate without samples. A Dilor LABRAM-1B multi-channel confocal micro spectrometer (Dilor, Lille, France) with 532 nm wavelength and 0.5 mW of incident excitation power was used to record the Raman spectra.

## 4. Conclusions

In summary, multifunctional hollow porous Fe_3_O_4_@N-C core-shell nanocomposites have been fabricated successfully by a simple self-template method, which involved the preparation of Fe_3_O_4_ nanospheres via the solvothermal method, in situ formation of dopamine coating and subsequent calcination in N_2_. Then, the Fe_3_O_4_@N-C nanocomposites were etched in hydrochloric acid solution by controlling the reaction time, and finally a series of hollow porous Fe_3_O_4_@N-C nanocomposites were acquired. The unique hollow porous core-shell structure with appropriate N-doped carbon coating can shorten the ion/electron transport channel, reduce the volume change during the repeated insertion/extraction of lithium ions and improve the structural stability. The typical Fe_3_O_4_@N-C-3 nanocomposites as an anode for LIBs exhibit excellent specific capacity, rate capacity and cycling performance. Meanwhile, Fe_3_O_4_@N-C nanocomposites also show expansive application prospects in SERS determination and the adsorption of pollutants in water. The removal rate of MO reached 98.65% after 100 min when Fe_3_O_4_@N-C-3 was used as the adsorbent and the Raman spectral response intensity of the thiram increased obviously on Fe_3_O_4_@N-C substrates. In the future, the electrochemical performance of a full battery containing Fe_3_O_4_@N-C anode materials, the faster and more selective adsorption of the samples towards organic pollutants in wastewater and the sensitivity of Fe_3_O_4_@N-C SERS substrates for detecting pesticide residues could be further investigated to explore the practical application possibility of products.

## Figures and Tables

**Table 1:** Contrast of the electrochemical performances of similar LIB anode materials.

Samples	Current Density mA g^−1^	Cycle Numbers	Capacity mA h g^−1^	Ref.
yolk–shell Fe_3_O_4_@C	150	20	1010	[34]
Fe_3_O_4_/C nanofibers	500	150	780	[20]
core–shell Fe_3_O_4_@C and Fe@C	50	40	1080	[35]
3D porous Fe_3_O_4_	1000	100	1382	[31]
porous Fe_3_O_4_/C microspheres	100	100	1180	[36]
hierarchical Fe_3_O_4_@NC	500	100	992	[37]
hollow porous Fe_3_O_4_@N/C	200	100	1772	This work

## Data Availability

Not applicable.

## Sample Availability

Samples of the compounds are not available from the authors.

## References

[1] Ye H., Zheng G., Yang X., Zhang D., Zhang Y., Yan S., You L., Hou S., Huang Z. (2021). Application of different carbon-based transition metal oxide composite materials in lithium-ion batteries. J. Electroanal. Chem..

[2] Wang X., Yang J., Zhou L., Song G., Lu F., You L., Li J. (2021). Rapid and ultrasensitive surface enhanced Raman scattering detection of hexavalent chromium using magnetic Fe_3_O_4_/ZrO_2_/Ag composite microsphere substrates. Colloids Surf. A Physicochem. Eng. Asp..

[3] Dehmani Y., Dridi D., Lamhasni T., Abouarnadasse S., Chtourou R., Lima E.C. (2022). Review of phenol adsorption on transition metal oxides and other adsorbents. J. Water Process Eng..

[4] Molaiyan P., Dos Reis G.S., Karuppiah D., Subramaniyam C.M., García-Alvarado F., Lassi U. (2023). Recent Progress in Biomass-Derived Carbon Materials for Li-Ion and Na-Ion Batteries—A Review. Batteries.

[5] Abdollahifar M., Molaiyan P., Lassi U., Wu N.L., Kwade A. (2022). Multifunctional Behaviour of Graphite in Lithium–sulfur Batteries. Renew. Sustain. Energy Rev..

[6] Yong L., Duan C., Jiang Z., Zhou X., Wang Y. (2021). CuO/rGO nanocomposite as an anode material for high-performance lithium-ion batteries. Mater. Res. Express.

[7] Dong H., Deng M., Sun D., Zhao Y., Liu H., Xie M., Dong W., Huang F. (2022). Amorphous Lithium-Phosphate-Encapsulated Fe_2_O_3_ as a High-Rate and Long-Life Anode for Lithium-Ion Batteries. ACS Appl. Energy Mater..

[8] Sun Y., Huang F., Li S., Shen Y., Xie A. (2017). Novel porous starfish-like Co_3_O_4_@nitrogen-doped carbon as an advanced anode for lithium-ion batteries. Nano Res..

[9] Yi Z., Lin N., Xu T., Qian Y. (2018). TiO_2_ coated Si/C interconnected microsphere with stable framework and interface for high-rate lithium storage. Chem. Eng. J..

[10] Gao Z., Yang Y., Qin J., Su Z. (2021). A core-shell porous MnO_2_/Carbon nanosphere composite as the anode of lithium-ion batteries. J. Power Sources.

[11] Jang J., Song S., Kim H., Moon J., Ahn H., Jo K., Bang J., Kim H., Koo J. (2021). Janus Graphene Oxide Sheets with Fe_3_O_4_ Nanoparticles and Polydopamine as Anodes for Lithium-Ion Batteries. ACS Appl. Mater. Interfaces.

[12] Kopuklu B., Kopuklu A., Yurum A., Kopuklu A. (2021). High stability graphene oxide aerogel supported ultrafine Fe_3_O_4_ particles with superior performance as a Li-ion battery anode. Carbon.

[13] Hao S., Li Q., Qu J., An F., Zhang Y., Yu Z. (2018). Neuron-Inspired Fe_3_O_4_/Conductive Carbon Filament Network for High-Speed and Stable Lithium Storage. ACS Appl. Mater. Interfaces.

[14] Zhang K., Zhang Q., Gao X., Chen X., Wang Y., Li W., Wu J. (2018). Effect of absorbers’ composition on the microwave absorbing performance of hollow Fe_3_O_4_ nanoparticles decorated CNTs/graphene/C composites. J. Alloys Compd..

[15] Zhang K., Zhang Q., Gao X., Chen X., Shi J., Wu J. (2018). Ellipsoidal Fe_3_O_4_@C nanoparticles decorated fluffy structured graphene nanocomposites and their enhanced microwave absorption properties. J. Mater. Sci. Mater. Electron..

[16] Wang J., Zhang M., Xu J., Zheng J., Hayat T., Alharbi N.S. (2018). Formation of Fe_3_O_4_@C/Ni microtubes for efficient catalysis and protein adsorption. Dalton Trans..

[17] Lin Q., Wang J., Zhong Y., Sunarso J., Tadé M.O., Li L., Shao Z. (2016). High performance porous iron oxide-carbon nanotube nanocomposite as an anode material for lithium-ion batteries. Electrochim. Acta.

[18] Bhattacharjya D., Park H., Kim M., Choi H., Inamdar S.N., Yu J. (2014). Nitrogen-Doped Carbon nanoparticles by flame synthesis as anode material for rechargeable lithium-ion batteries. Langmuir.

[19] Peng C., Zhou S., Zhang X., Zeng T., Zhang W., Li H., Liu X., Zhao P. (2018). One pot synthesis of nitrogen-doped hollow carbon spheres with improved electrocatalytic properties for sensitive H_2_O_2_ sensing in human serum. Sens. Actuators B Chem..

[20] He J., Zhao S., Lian Y., Zhou M., Wang L., Ding B., Cui S. (2017). Graphene-doped carbon/Fe_3_O_4_ porous nanofibers with hierarchical band construction as high-performance anodes for lithium-ion batteries. Electrochim. Acta.

[21] Tran T., Phan T., Nguyen D., Nguyen T., Nguyen D., Vo D., Bach L., Nguyen D. (2021). Recyclable Fe_3_O_4_@C nanocomposite as potential adsorbent for a wide range of organic dyes and simulated hospital effluents. Environ. Technol. Innov..

[22] Xu J., Li X., Wang Y., Guo R., Shang S., Jiang S. (2019). Flexible and reusable cap-like thin Fe_2_O_3_ film for SERS applications. Nano Res..

[23] Yang Y., Gao X., Yang S., Shen Y., Xie A. (2021). Synthesis and superior SERS performance of porous octahedron Cu_2_O with oxygen vacancy derived from MOFs. J. Mater. Sci..

[24] Liu L., Pan F., Liu C., Huang L., Li W., Lu X. (2018). TiO_2_ nanofoam–nanotube array for surface-enhanced Raman scattering. ACS Appl. Nano Mater..

[25] Xu J., Zhang J., Wang C. (2017). Carbon encapsulated Fe_3_O_4_ nanospheres with high electrochemical performance as anode materials for Li-ion battery. Int. J. Appl. Ceram. Technol..

[26] Han F., Ma L., Sun Q., Lei C., Lu A. (2014). Rationally designed carbon-coated Fe_3_O_4_ coaxial nanotubes with hierarchical porosity as high-rate anodes for lithium ion batteries. Nano Res..

[27] Jiang Z., Jiang Z. (2014). Fabrication of Nitrogen-Doped Holey Graphene Hollow Microspheres and Their Use as an Active Electrode Material for Lithium Ion Batteries. ACS Appl. Mater. Interfaces.

[28] Lei C., Han F., Sun Q., Li W., Lu A. (2014). Confined nanospace pyrolysis for the fabrication of coaxial Fe_3_O_4_@C hollow particles with a penetrated mesochannel as a superior anode for Li-ion batteries. Chemistry.

[29] Dang R., Jia X., Liu X., Ma H., Gao H., Wang G. (2017). Controlled synthesis of hierarchical Cu nanosheets@CuO nanorods as high-performance anode material for lithium-ion batteries. Nano Energy.

[30] Wang L., Yu Y., Chen P., Zhang D., Chen C. (2008). Electrospinning synthesis of C/Fe_3_O_4_ composite nanofibers and their application for high performance lithium-ion batteries. J. Power Sources.

[31] Wu H., Du N., Wang J., Zhang H., Yang D. (2014). Three-dimensionally porous Fe_3_O_4_ as high-performance anode materials for lithium–ion batteries. J. Power Sources.

[32] Zhou G., Wang D., Li F., Zhang L., Li N., Wu Z., Wen L., Lu G., Cheng H. (2010). Graphene-Wrapped Fe_3_O_4_ Anode Material with Improved Reversible Capacity and Cyclic Stability for Lithium Ion Batteries. Chem. Mater..

[33] Cherian C., Sundaramurthy J., Kalaivani M., Ragupathy P., Kumar P., Thavasi V., Reddy M., Sow C., Mhaisalkar S., Ramakrishna S. (2012). Electrospun α-Fe_2_O_3_ nanorods as a stable, high capacity anode material for Li-ion batteries. J. Mater. Chem..

[34] Zhao Y., Li J., Wu C., Ding Y., Guan L. (2012). A Yolk-Shell Fe_3_O_4_@C Composite as an Anode Material for High-Rate Lithium Batteries. ChemPlusChem.

[35] Su L., Zhong Y., Zhou Z. (2013). Role of transition metal nanoparticles in the extra lithium storage capacity of transition metal oxides: A case study of hierarchical core–shell Fe_3_O_4_@C and Fe@C microspheres. J. Mater. Chem. A.

[36] Deng W., Ci S., Li H., Wen Z. (2017). One-step ultrasonic spray route for rapid preparation of hollow Fe_3_O_4_/C microspheres anode for lithium-ion batteries. Chem. Eng. J..

[37] Jiang F., Liu Y., Wang Q., Zhou Y. (2017). Hierarchical Fe_3_O_4_@NC composites: Ultra-long cycle life anode materials for lithium ion batteries. J. Mater. Sci..

[38] Nsabimana A., Kitte S., Wu F., Qi L., Liu Z., Zafar M., Luque R., Xu G. (2018). Multifunctional magnetic Fe_3_O_4_/nitrogen-doped porous carbon nanocomposites for removal of dyes and sensing applications. Appl. Surf. Sci..

[39] Zhao D., Lin K., Wang L., Qin Z., Zhao X., Du K., Han L., Tian F., Chang Y. (2020). A physical approach for the estimation of the SERS enhancement factor through the enrichment and separation of target molecules using magnetic adsorbents. RSC Adv..

[40] Li L., Zhao A., Wang D., Guo H., Sun H., He Q. (2016). Fabrication of cube-like Fe_3_O_4_@SiO_2_@Ag nanocomposites with high SERS activity and their application in pesticide detection. J. Nanoparticle Res..

[41] Yao C., Gao X., Liu X., Shen Y., Xie A. (2021). In-situ preparation of Ferrero (R) chocolate-like Cu_2_O@Ag microsphere as SERS substrate for detection of thiram. J. Mater. Res. Technol..

[42] Liu Z., Wang Y., Deng R., Yang L., Yu S., Xu S., Xu W. (2016). Fe_3_O_4_@Graphene Oxide@Ag Particles for Surface Magnet Solid-Phase Extraction Surface-Enhanced Raman Scattering (SMSPE-SERS): From Sample Pretreatment to Detection All-in-One. ACS Appl. Mater. Interfaces.

